# Italian Adaption of Self-Perceived Employability Scale: Psychometric Properties and Relations with the Career Adaptability and Well-Being

**DOI:** 10.3390/bs10050082

**Published:** 2020-04-27

**Authors:** Ernesto Lodi, Andrea Zammitti, Paola Magnano, Patrizia Patrizi, Giuseppe Santisi

**Affiliations:** 1 Department of Humanities and Social Sciences, University of Sassari, Via Roma 151, 07100 Sassari, Italy; patrizi@uniss.it; 2 Department of Educational Sciences, University of Catania, Via Biblioteca 4, 95124 Catania, Italy; andrea.zammitti@phd.unict.it (A.Z.); gsantisi@unict.it (G.S.); 3 Faculty of Human and Social Sciences, Kore University, Cittadella Universitaria, 94100 Enna, Italy; paola.magnano@unikore.it

**Keywords:** employability, self-perceived employability scale, career paths, well-being

## Abstract

The recent transformation of the workplaces and labor market, characterized by rapid technological changes, social and economic instability, has greatly influenced the construction of people’s career paths. These paths cannot be viewed more as linear, but multifaceted and unstable. In organizational context, the psychological contract has changed from long term to short term. In this scenario, the construct of employability becomes central: people need to maintain and improve their ability to be attractive to the labor market to get or keep a job. The study presents the adaptation of the Self-Perceived Employability Scale to the Italian context. The participants are 660 Italian workers. The instruments used to verify the concurrent validity of the scale were the Employability Scale, the Flourishing Scale, the Satisfaction With Life Scale, the Organizational Satisfaction Questionnaire and the Career Adapt-Abilities Scale. Results showed good psychometric properties of the Italian version in terms of internal consistency, construct and concurrent validity, with significant correlations with all the other measures. The CFA highlights some dissimilarity in the scale’s structure compared to the UK version, probably due to cultural differences among the samples.

## 1. Introduction

The term employability refers to the probability of obtaining or keeping a job [1,2]; employability is also defined as the ability to be employed, that is: the ability to obtain an initial job, to keep a job being able to manage one’s transitions in the world of work [3]. Rothwell and Arnold [4] define employability as the ability to get the job you want or to keep your job.

Initially, this construct was studied in relation to unemployed people, but today it is considered important also for the employed [5]. Indeed, today, the work is different than in the past. In fact, the workers now have to face a turbulent career environment characterized by uncertainty and insecure working conditions, requiring frequent transitions. In surviving in this working environment, workers are expected to change themselves and actively construct their professional projects, developing psychosocial resources [6,7], including self-perceived employability [2].

Within this new scenario, linear career forms have given way to other career forms [8,9,10], the psychological contract has become short term rather than long term [9,10], and more and more often, knowledge, skills, education, etc., are placed at the center of economic development processes [11]. For these reasons, employers cannot guarantee job security; therefore, they should offer continuous skills development with the aim of improving attractiveness within the labor market [12].

Given these recent transformations of the workplaces and labor market that have influenced people’s career paths construction, the concept of employability is a core psychosocial resource that provides a general scan as it combines all possible individual and structural factors and their interrelationships; and it may present a first indication as to whether the labor market position is perceived as relatively weak or strong [13].

For these reasons, the study aims to adapt to the Italian context a reliable and multidimensional instrument to assess the employability at workers’ level: the Self-Perceived Employability Scale [4]. The research is focused on the assessment of employability through this instrument and its psychometric properties.

After a literature analysis, the main aims that this study will want to achieve are: (a) to verify the structure and reliability of the Italian version of instrument through an EFA and Cronbach’s alpha; (b) to test a latent factorial structure through Confirmatory Factorial Analysis (CFA); (c) to confirm the validity of the scale scores through the Employability Scale [14], SWLS-Satisfaction With Life Scale [15], Flourishing Scale [16] and the Career Adapt-Abilities Scale as external criteria. In particular, we choose the Career Adaptability because the ability to adapt to new situations is a component of employability [2], and the measures of domain-specific and general well-being because the employability has showed, in many studies, its relationships with these kind of outcomes [7,17].

## 2. Literature Review 

### 2.1. Components of Self-Perceived Employability

According to Fugate et al. [2], the components of employability are: career identity, personal adaptability and social and human capital. These dimensions together constitute the employability construct. Career identity refers to how individuals define themselves in a particular work context; career identity provides some cognitive patterns that influence behavior [18]. Personal adaptability refers to the ability to adapt to new situations and contributes to career success because individuals with high levels of personal adaptability are more attractive in sectors of work that are constantly evolving [19,20]. Social and human capital is the third dimension that constitutes employability. Social capital refers to social networks. People with developed social capital are better able to use formal and informal networks to look for work; human capital refers to a number of variables that influence an individual’s career advancement: age and education [21], work and training experience [22], assignment work and organizational [23], emotional intelligence [24] and cognitive skills [25].

Hillage and Pollard [3] argue that four elements of employability can be identified: assets, which include knowledge (what people know), skills (what they can do with their knowledge) and attitudes (how they do it); deployment, which covers career management skills, job search skills and strategic approach, that is, the ability to adapt to the job market and be realistic about the opportunities offered; presentation, or the ability to present one’s employability (presentation of CVs, academic and professional qualifications, etc.); personal and external circumstances, which include, for example, a person’s disability or family status or the demand for work in the labor market.

Rothwell and Arnold [4] point out that employability is made up of both individual attributes but also external factors to the individual. From the point of view of attributes, they have identified that the knowledge and skills of the individual fall into this category [3], but also their ability to learn [26,27], the individual’s mastery of managing his career and looking for work [3], professional knowledge [28], resilience [29,30,31] and self-efficacy [32]. From the point of view of factors external to the individual, we must mention: the labor market [3,27,31,33] and the demand for occupation [34]. Therefore, in literature, the distinction between internal and external employability is widely debated.

Self-efficacy and self-perception have effects on employability [35]. Perceived employability refers to the individual’s perception of the possibility of obtaining and keeping a job [4,36,37].

Vanhercke and colleagues [13] highlight five important aspects in this definition. The fact that it is a subjective assessment, therefore, similar individuals may have different perceptions of their employability; employability concerns employment “possibilities”, considering both personal and work-related factors and organization, support in career development and society [4,38,39,40,41,42]; employability is relevant for students [3,43], employed individuals [4,36] and unemployed individuals [44,45], as it is important for exploring the work market, finding employment and managing transitions [46]; perceived employability refers to “employment” opportunities; the focus concerns both the quantity and quality of jobs, meaning the number of available opportunities and the type of work.

### 2.2. Career Adaptability and Self-Perceived Employability

Career adaptability is a key concept of the broader career-building theory developed by Savickas [47], which explains how individuals interact with the environment to produce important changes in their lives. Savickas [48] defined career adaptability as “… the readiness to cope with the predictable tasks of preparing for and participating in the work role and with the unpredictable adjustments prompted by the changes in work and work conditions” [48] (p. 254). Career adaptability is made up of four dimensions: the propensity to worry positively for the future (Career Concern), the conviction that the future is controllable (Career Control), the curiosity about the professional world (Career Curiosity) and the belief in the self to achieve one’s career goals and solve problems (Career Confidence) [47]. According to Savickas [49], career adaptability represents an important resource that can support employability.

There are many studies that have shown that career adaptability is positively correlated with various variables related to the individual, career and work: high levels of career adaptability are related to feelings of self-efficacy in finding a job [50], professional satisfaction [51], ability to manage transitions [52,53], greater security in achieving their goals [54] and greater life satisfaction [55,56]. Furthermore, in a meta-analysis conducted by Rudolph, Lavigne and Zacher [57], career adaptability was significantly associated with employability in several studies. For example, Coetzee, Ferreira, and Potgieter [58] have shown that there are positive relations between the employability capacities and career adaptability dimensions in employed adults. De Guzman and Choi [59] also observed significant relationships between career adaptability and employability. Atitsogbe et al. [60] found that career adaptability, along with self-efficacy, is positively related to self-perceived employability; the latter is positively related to business intentions. Udayar and colleagues [61] showed that career adaptability is a mediator in the relationship between trait emotional intelligence and self-perceived employability.

### 2.3. Job Satisfaction and Self-Perceived Employability

Job satisfaction is defined as “a pleasurable or positive emotional state resulting from the appraisal of one’s job” [62] (p. 1304). Other authors also confirm this definition and indicate that job satisfaction is an affective orientation that the individual has towards their work [63,64,65,66]. This construct has been studied above all since the 1950s and many authors have studied its link with other dimensions.

Some studies have tried to understand what the antecedents are and the consequences of job satisfaction.

The antecedent of job satisfaction can be linked to the characteristics of the job or to individual characteristics. The characteristics of the work refer to the role, the workload, the control over one’s work, the schedules, the family-work conflict, the way in which human resources are managed or relationships in the workplace [67,68,69,70,71,72]. The individual characteristics may be the locus of control and negative affectivity [73].

Job satisfaction is related to employee loyalty [74], work climate [75], psychosocial work climate [76], diversity climate [77] and self-efficacy [78,79].

Some research has investigated the relationship between employability and job satisfaction: for example, Karren and Gowan [80], in a longitudinal research conducted on people who have lost their jobs, have shown that employability predicted well-being and job satisfaction. Employability has to do with the value of the person in the job market; for this reason, different studies [3,39,81] have shown that people who have higher levels of employability also have higher levels of job satisfaction. This is because these people have more ability to get the job they want and find it more satisfying. In a study of 414 Chinese adults, Ngo, Liu and Cheung [82] found that perceived employability correlates positively with job satisfaction and mediates the effect of self-efficacy on job satisfaction.

### 2.4. Well-Being and Self-Perceived Employability

The literature analyzing the relationship between self-perceived employability and well-being is not large, but both cross-sectional [1,83,84] and longitudinal studies [36] established that employability is related to employees’ general health, psychological well-being, domain-specific (job) and general well-being. According to De Cuyper et al. [83], employability may enhance employees’ sense of control of one’s career, which, consequently, relates to well-being [2,85]. Furthermore, workers with a higher level of employability may believe that there are potentially better alternatives to adopt in job search behavior. This could lead these workers to the possibility of finding good quality jobs, which have been shown to promote higher well-being levels. Therefore employability is a key factor especially during times of economic recession, as it provides workers with a wide range of options and choices that could make them less vulnerable [86]. Furthermore, Trevor [87] and Pfeffer [88] theorized that employable persons, when involved in a not satisfying or under-rewarding job, are more likely to quit it, rather than continuing to feel locked in an unpleasant job [86]. Silla et al. [89] demonstrated that employability was a significant predictor of workers’ well-being levels in the case of a career without borders [39,90].

### 2.5. Review of the Existent Measures of Employability 

Employability has been evaluated through two different types of measures: the objective ones, which take into account socio-demographic variables such as education [91] and self-assessed measures, that give importance to self-perceptions as individuals act on the basis of their perceptions rather than on objective reality [92]. For example, Groot and Maassen Van den Brink [91], in their study, considered variables such as education level or wages and found that the training of individuals increases employability and is not related to wages. Thiessen and Looker [93], instead, considered the number of transitions made.

Other studies, however, are more consistent with the psychological approach to employability. Gunawan, Creed and Glendon [94] considered the perspective of Perceived Future Employability that is the representation of one’s professional self at the end of the studies. The future self refers to the individuals’ representations of what they might become [95]. The future self can become the basis for anticipating future events, setting goals, planning, and exploring the possible options [96]. From this perspective, the authors created a scale that measures perceived future employability. The tool consists of 24 items on a 6-level Likert scale, from 1 = strongly disagree to 6 = strongly agree. The authors also demonstrated the positive correlation of the construct with career ambition and university commitment, and the negative correlation with professional hardship [94].

Misra and Mishra [97], citing Robinson’s [98] perspective, which defines employability skills as “those basic skills necessary for getting, keeping and doing well on a job, and more so are skills that can be taught” [98] (p. 1), have developed a scale for assessing the employability skills. The authors formulated an initial set of 60 items on 5-point scale ranging from “1” strongly disagree to “5” strongly agree. Following the statistical analyses, the factors that emerged are six: (1) the enhancement of skills, which refers to the fact that people make efforts to add skills and support their professional growth; (2) task-orientation, referring to the willingness to take on new positions; (3) being a blue-eyed boss boy, who refers to being known to his superiors and professionals thanks to his experiences; (4) professional networking, which reflects how much an individual receives help from other people related to his or her professional network to get new jobs or to be included in new projects; (5) concern for time and (6) love for challenge.

In their study aimed to better understand the phenomenon of job insecurity, Lo Presti and Nonnis [14] used a scale to evaluate the employability that was initially proposed during a personal communication by Isabelle Hansez (University of Liege). The scale is made up of five items and asks the participant to answer how likely he is to find certain situations (for example, finding a job outside his company) on a Likert scale ranging from 1 (no probability) to 5 (sure, 100% probability). This measure has also proved effective in other studies [99,100].

Rothwell, Herbert and Rothwell [101] developed a measure of the expectations and self-perceptions of employability of university students. The authors examined the employability from the perspective of individuals, “that is, what individuals seeking a particular type of work believe their chances of success are, and what factors influence their perceptions” [101] (p. 1). Inspired by the definition already of Hillage and Pollard [3], the authors suggest that in university, students perceived employability as defined as the ability to obtain a job appropriate to one’s qualification [101]. In addition to considering the internal and external dimensions of employability, the authors added two new dimensions to their study: (1) the brand of the university and (2) the impact of the student’s field of study. Indeed, Murray and Robinson [102] have shown how recruiters refer to a limited number of universities; this could have an impact on the perception of employability. Furthermore, according to the field of study, the request for work changes and students try to anticipate what could be the future trend in the professional attractiveness of individual subjects [103]. The final scale that the authors developed is made up of 16 items to which participants respond on a Likert-type scale from strongly disagree (1) to strongly agree (5). This scale has shown good reliability in different contexts [104,105,106,107,108,109,110] including the Italian context [111].

Rothwell and Arnold [4] resumed the 16-item scale and developed another version keeping 11 items. In constructing the initial 16 items, the authors took into consideration four quadrants: a quadrant “a” which represents the assessment that the individual believes that the organization attributes to him regarding its usefulness; a quadrant “b” which concerns the way in which the organization evaluates the occupation or the professional group; a quadrant “c” which refers to the self-perception that the individual has of his own value in the labor market; and a “d” quadrant which refers to the perception of the external labor market. Starting from these four quadrants, the authors generated 16 items and took care to include elements reported in the literature: (1) skills and behaviors that contribute to effective performance [3,28]; (2) resilience [31]; (3) contacts that can provide information and support [2]; and (4) job search skills and job market knowledge [3].

Research participants were asked to answer each of the 16 questions on a 5-point scale, from strongly disagree (1) to strongly agree (5).

Following the subsequent statistical analyses, the Self-Perceived Employability Scale looks like a tool consisting of 11 items to 5-point Likert-type scale (from 1 disagree to 5 strongly agree) to assess perceived employability as a unitary construct or two factorial components. The instrument evaluates the two sub-dimensions: (a) Internal Employability: item 1-2-3-4-7; (b) External Employability: item 5-6-8-9-10-11 (see Appendix A). The authors suggested to be cautious treating self-perceived employability as a mono-dimensional construct because the two-dimensional solution created an appropriate division between elements that made up employability within and outside the organization. Finally, the authors, evaluating the goodness of the instrument, claimed that “the measure very successfully distinguished employability from professional commitment, and fairly successfully from career success” [4] (p. 23).

## 3. Aim of the Study

The aim of the study is verifying the psychometric properties of the Italian version of the Self-Perceived Employability Scale, showing its factorial structure, reliability, construct and concurrent validity.

## 4. Materials and Methods

### 4.1. Translation

The Self-Perceived Employability Scale’s items were translated into Italian according to the translation/back-translation process. The items were submitted to a small number of workers in order to verify understandability of the items. After this step, no modifications were made to the items and this version was then administered in the main study. The scale Italian/English is available in Appendix A.

### 4.2. Participants

The main study was conducted on 660 Italian workers (Mean Age = 40.76; S.D. = 11.18) involved in private and public jobs in small and medium organizations (SMOs). The women were more represented than men (F = 366; 55.5%; M = 294; 44.5%). The 56.7% (n = 374) of the participants were married, and the percentage of education level were thus distributed: 6.5% (n = 43) junior high school degree; 41.4% (n = 273) high school graduation; 34.4% (n = 227) university degree; 17.7% (n = 117) post-graduate degree. The respondents lived in different Italian regions. They work in public (43%), private (52.6%) and non-profit (4.4%) organizations. More than half of them have permanent contracts (59.2%); the remaining part have fixed-term contracts (17.4%) and other forms of contracts (23.4%). The majority of them (85.5%) declared that the actual job is consistent with his/her professional interests and competences. About half of them have worked in their organization for more than ten years (44.8%).

The recruitment of the participants used the convenience sampling; the participants were enrolled on a voluntary basis in an online survey and they were free to abandon their participation at any moment.

### 4.3. Measures

The instruments used with Self-Perceived Employability Scale were:

1. Satisfaction with Life Scale (SWLS) [15]. It is a mono-dimensional Likert scale composed of five items (from 1 “strongly disagree” to 7 “strongly agree”) that evaluates people’s overall life satisfaction. The Cronbach’s alpha is 0.85; in our study was 0.88.
2. Employability Scale [14], a 5-item Likert scale (1 = no probability; 5 = 100% probability/certain) that measure the individual perception about the probability to get a new job. The Cronbach’s alpha is 0.90; in our study was 0.93.
3. Flourishing Scale [112] is a 5-item Likert scale from 1 (strongly disagree) to 7 (strongly agree) that evaluates in one dimension the meaning and purpose in life. The Cronbach’s alpha is 0.91; in our study sample was 0.88.
4. Organizational Satisfaction Questionnaire (QSO) developed by Cortese [113] that evaluates the job satisfaction. It consists of 20 items in a 7-point Likert scale, that consider multiple aspects of job satisfaction such as autonomy, benefits, career, internal communication, working conditions, prestige, work/leisure balance, organization, relationship, remuneration, recognition, safety in the workplace, and type of activity. In our study, we calculated a single global factor and the Cronbach’s alpha was 0.94.
5. Career Adapt-Abilities Scale [114], Italian Form by Soresi, Nota and Ferrari [115]. The scale consists of 24 items (Likert scale from 1 “not strong” to 5 “strongest”). The instrument provides a general measure of career adaptability and four sub-dimensions: Concern, Control, Curiosity and Confidence. The Cronbach’s alpha values for the subscales are: 0.74, 0.80, 0.87, and 0.84 respectively. In our sample, Cronbach’s alpha was 0.90, 0.87, 0.90, and 0.91 respectively.

### 4.4. Procedure and Data Analysis

The survey followed the ethical rules of the Italian Psychological Association and the ethical commission of the universities involved approved it. All workers were asked to fill out an anonymous questionnaire “about their experience as a worker”. To reach all workers, we chose an online survey, even if this choice can produce bias about the response rate. Workers were free to answer the survey and they can decide to stop their participation at any moment. The response rate was 17% of all workers; this rate, considering Nulty’s sampling criteria [116] is good according to “liberal conditions”, but not good enough according to “stringent conditions”. Therefore, the workers can decide if they want to report their name and surname in order to obtain a report about their results. The Self-Perceived Employability Scale was administered after some questions on demographic data (sex, age, job, marital status, education level).

The software used to process the data was IBM SPSS Statistics for Windows, Version 25.0. Armonk, NY: IBM Corp., and CFA was conducted by LISREL, Version 8.80 [117] with the maximum likelihood method (ML estimation) [118]. For the confirmatory factorial analysis, we considered the χ^2^ to verify the general adequacy of the model in fitting data. Since a significant χ^2^ value rejects the null hypothesis that the model fits in the population, a good solution fits the data when χ^2^ is non-significant (*p* > 0.05; [119]). The chi square/df ratio is another index almost used in the CFA literature, with <3 good values and <5 acceptable values. Generally, the χ^2^ test is not sufficient to test model goodness of fit because it is sensitive to the sample size [120]. Consequently, to verify the fit indices in structural equation models, we refer to the combined use of a Comparative Fit Index (CFI), Root Mean Square Error of Approximation (RMSEA) and the Standardized Root Mean Square Residual (SRMR). CFI acceptable or good values of fit are between 0.95 and 1 [119]. Good values of the Root Mean Square Error of Approximation (RMSEA) are lower than 0.05 [121], even if the limit of <0.08 can be considerate as acceptable [119], and in a smaller sample, according to Browne and Cudeck [122], we can also consider as acceptable values <0.09. The Standardized Root Mean Square Residual (SRMR) acceptable value is <0.08 [121,122].

To compare different models, we use the Akaike Information Criterion (AIC) [123]: a lower AIC value indicated a superior model fit compared with models with higher values. To evaluate the internal consistency, we used the Cronbach’s alpha index. To evaluate the alpha’s values, we used the following criteria: <0.60 not acceptable; between 0.60 and 0.70 acceptable; >0.70 good; >0.80 very good [124,125]. In addition, based on CFA results, we used composite reliability [126] and AVE (average variance extracted) [127] to evaluate the reliability of the model. The concurrent validity was computed: (a) correlating the scores of Self-Perceived Employability Scale with the scores of Employability Scale [14], Career Adapt-Abilities Scale [114], Flourishing Scale [112], SWLS [15] and Organizational Satisfaction Questionnaire [113] and (b) through multiple regressions with overall and domain-specific well-being measures as outcomes and Self-Perceived Employability dimensions as predictors.

## 5. Results 

Descriptive statistics (Table 1) revealed the normal distribution of all the items with skewness and kurtosis values from 0.07 to −0.94 (except item 7 with skewness −0.98 and kurtosis 1.57). The mean range is from 3.16 to 3.98 and SD range is from 0.86 to 1.22.

Since the values of skewness and kurtosis was not between 1 and −1 and in a previous exploratory factorial analysis (EFA) conducted, the item 7 showed communality value below 0.20 and significant cross-loadings on all the factors, we decided to eliminate item 7 in the Italian version.

The first step was choosing latent factorial structure of the remaining 10 items for the Italian sample using the exploratory factorial analysis and providing evidence about the internal consistency using the Cronbach’s alpha indices. Given the possible differences that could influence the structure of the two scales (original version and the Italian adapted version), before proceeding to the confirmatory factorial analysis, we decided to carry out an exploratory factorial analysis. Therefore, a translated test must be treated as a test developed from scratch to give a complete documentation of its structure, reliability and the various validity forms [128].

The EFA was conducted with Principal Axing Factoring method and Promax rotation. The Kaiser-Meyer-Olkin (KMO) was 0.82 and the Bartlett’s test of sphericity was significant (*p* < 0.001), indicating the adequacy of the data for the factorial analysis. Communality values were ranged between 0.31 (item 4) and 0.87 (item 5). The factorial analysis extracted three factors (Table 2) with 51.03% variance explained and eigenvalues > 1. Parallel analysis was applied through using the equations by Keeling [129] and by Lautenschlager and colleagues [130] and the results confirmed the three dimensions of Italian adaptation. Looking at factor loadings (>0.40), the three factors’ structure showed a good convergent validity. There are not significant cross-loadings indicating a good discriminative validity. We named the first factor *Future perspective* because in the items, the dimension of future perspectives about work and career path is prevalent. We named the second factor *Ability evaluation* because in the items, the dimension of skills and the thought about retraining is relevant. We named the third factor *External Work Opportunities* because in the items, the comparison between current conditions and opportunities with others imagined in a potential external organization is relevant.

The factors are inter-correlated (*p* > 0.001): Factors 1-2, r = 0.565; Factors 1-3, r = 0.450; Factors 2-3, r = 0.464. The three subscales have a normal distribution as presented in Table 3, where reported also are the Cronbach’s alpha values as indicators of internal consistency. The values reveal a good internal reliability for the factors. None of the items, if deleted, improves the Cronbach’s alpha of its own subscale. The homogeneity and consistency of the three sub-dimensions was demonstrated also by the inter-item correlations (>0.20) and item-total correlations (>0.45).

A CFA was then conducted on the 10 items retained after the exclusion of the item 7 (see Appendix A). The χ^2^ value is significant, but usually it happens because of the sample dimension. The chi square/df ratio is <5 indicating an acceptable value (χ^2^ = 155.49; df = 32). The other fit indices are good: CFI = 0.97; RMSEA = 0.08; SRMR = 0.05. Comparing the hypothesized model with a model with one factor (all items loading on a single factor) and with two factors (external and internal employability from the original version) revealed that the three factors model provided a better fit for the data in all the CFA fit measures (3-factor model: χ^2^_(32)_ = 155.49; CFI = 0.97; RMSEA = 0.08; SRMR = 0.05; AIC = 215.04; 1-factor model: χ^2^_(35)_ = 628.52; CFI = 0.84; RMSEA = 0.17; SRMR = 0.10; AIC = 759.79; 2-factor model: χ^2^(_34)_ = 449.53; CFI = 0.89; RMSEA = 0.14; SRMR = 0.09; AIC = 518.55. The differences were significant according to a comparison of the models’ χ^2^ values and degrees of freedom: 3-factors/1-factor: Δχ^2^_(3)_ = 473.63 (*p* < 0.001); 3-factors/2-factors: Δχ^2^_(2)_ = 294.04 (*p* < 0.001). Using the ML estimation method and considering that the values of the indices are below the acceptable parameters, these results suggested that the one or two factor original version structure could not be confirmed in the Italian adaptation. Therefore, the need of change to the original structure of the scale emerges because it is not applicable to the Italian cultural context.

Analyzing the model’s validity and reliability, we observed that all the items load significantly on the latent variables (factor loadings range 0.55–1.03; t > 2.58), indicating a good convergent validity. The constructs showed good values of composite reliability (>0.65) and acceptable values of average variance extracted (>0.40).

Concurrent validity: The three subscales showed a positive and significant relationship (*p* < 0.001) with Employability Scale having correlations coefficient medium-low or medium (Employability Future Perspective: r = 0.17; Employability Ability Evaluation: r = 0.35; Employability External Work Opportunities: r = 0.42). Therefore, the three subscales showed positive and significant relationships with sub-dimensions of Career Adaptability and the Well-Being indices with correlations coefficient medium-low or medium (Table 4).

Multiple regressions (*method enter*) were conducted to verify the amount of variance explained by the Italian version of instrument on the scores of domain-specific and overall satisfaction (Table 5). The variance explained by the three sub-dimensions of Self-Perceived Employability is 15% on Life Satisfaction, 17% on Flourishing and 22% on Job Satisfaction. The significant predictors are all the three factors on Flourishing, the two sub-dimensions of Future Perspective and External Work Opportunities Employability on Life Satisfaction, and the dimension of Future Employability on Job Satisfaction.

## 6. Discussion

The Italian adaptation of Self-Perceived Employability has revealed good psychometric properties in terms of reliability and factor structure.

After having tested the original factor structure on the Italian sample, the non-satisfying results obtained suggested to us to explore new factorial solutions; so, we decided to delete the item 7, that showed a low communality value and cross-loadings on all the factors; then we conducted an EFA with the remaining 10 items (see Appendix A), obtaining a three-factor solution, confirmed by a CFA, whose fit indexes are significantly better than the original version of the scale. Analyzing the content of the items, we have named the factors as follows: *External Work Opportunities*, that is, similar to the external dimension of the English version; *Ability Evaluation*, that is, similar to occupational expertise in Van Der Heijde and Van Der Heijde’s definition [131]; moreover, De Vos and De Hauw [132] underlined that the employee’s know-how and skills are included in various definitions of employability; *Future Perspective,* that is, present in Fugate et al.’s [2] (2004) definition of employability, and in Van Der Heijde and Van Der Heijde [131] (p. 454) who identify a dimension called “Anticipation and optimization” […] that “entails preparing for future work changes in a personal and creative manner in order to strive for the best possible job and career outcomes” [133,134]. Rothwell and Arnold [4] themselves, in their conceptualization of employability, include the future-oriented perspective as the individual’s capacity to proactively face the challenges of the labor market.

The Italian version of the instrument has revealed good properties in terms of concurrent validity. All the correlations with the Employability Scale were positive and significant, even if the correlations coefficients are medium-low or medium. This data could be explained by the multidimensional nature of the Self-Perceived Employability Scale compared to the one-dimensional nature of the Employability Scale. As we expected, the relations between Career Adaptability, Well-Being indices and the Self-Perceived Employability were confirmed. These results are coherent with previous studies that have underlined the relationship among these variables [39,57,81]. Additionally, the regression’s results seem consistent with the literature on the topic. In fact, we have a percentage of variance explained below on life satisfaction which is a more general dimension linked to subjective well-being [16]. The variance explained increases with Flourishing, a dimension more related to the full development and utilization of one’s skills, competences and talents [135]. In this perspective, the higher variance explained on the variable more linked to domain-specific context (Job Satisfaction), that concerns more closely the work activities, seems to be consistent.

Since the concept of skills is far from the subjective well-being dimension (more related to the emotional states and personality traits), it seems consistent that the sub-dimension of the Ability Evaluation does not appear among the significant predictors of life satisfaction; whereas it appears as a predictor on Flourishing that is more related to the concept of psychological well-being. Unexpectedly, however, it does not appear as a predictor on Job Satisfaction. This could be due to the instrument we have chosen for the study: the job satisfaction is measured through numerous organizational and contextual variables (i.e., benefits, internal communication, working conditions, prestige, work-life balance, organization, relationship, remuneration, safety in the workplace) rather than on worker’s characteristics and skills.

The dimension of Future Perspective appears in all the models as a significant predictor. This demonstrates the importance of the future dimension in addressing the new risks associated with the current socio-economic context. Furthermore, numerous studies have demonstrated the relationship between temporal perspective (in particular, the future) and subjective, psychological and specific-domain well-being. Numerous previous studies have found significant relationships between Future Perspective and subjective well-being [136,137,138]; as underlined by Drake et al. [139], individuals with a more future-oriented outlook are able to anticipate positive outcomes [140]. Moreover, in the review reported by Bajec [141], the relationship between Future Perspective and Job Satisfaction has been previously demonstrated [142,143].

The dimension of External Work Opportunities appears as predictor on Life Satisfaction and Flourishing, but not on Job Satisfaction. Maybe because Job Satisfaction is more related to the actual organization where the workers live day-by-day rather than focused on a hypothetical external organization. Similar results have been found by De Cuyper and colleagues [1], who explained that perhaps job satisfaction is dependent by specific features of the present job and less related to the job opportunities in general or to the career as a whole. However, the relationship between Self-Perceived Employability and Job Satisfaction is not definitive and further research is needed [1].

## 7. Conclusions and Limitations

The results of our study indicated good psychometric properties of the Italian version of Self-Perceived Employability Scale in terms of internal consistency, construct and concurrent validity, with significant correlations with all the other measures. The CFA highlights some differences in the scale’s structure with respect to the original version that have showed one or two factor dimensionalities.

In times of instability of the labor market and unpredictability of the career environment, the perception of a greater job insecurity and occupational uncertainty may reduce the range of workers’ career objectives and aspirations [144]. The traditional concept of career was previously defined through a linear progression in a single or a limited number of organizations; in the 21st century, the careers are characterized by the experience of being self-managed, protean, and boundaryless, comprising many positions with multiple organizations [2]. Surviving to this instable career environment leads the workers to become competent to manage changes in themselves and in their contexts [20,145]. The employability, conceptualized as a psychosocial individual resource, can be considered “a key factor balancing the demands related to the changing nature of work, with its individualized responsibility and increased demands for flexibility” [146,147,148,149] (p. 218). Employability enhances in people the possibility to feel capable of a change in employment, should it be needed [150,151]. This possibility is linked to the individual perception as employable, so that a flexible context with unpredictable working conditions and job uncertainty cannot be contemplated as threatening, as it would be if people have lower perceptions of employability [149]. As highlighted by Rothwell et al. [101], employability in the career intervention context is often used as an *umbrella term* that holds a variety of tools and techniques aimed to strengthen the acquisition of individual skills and resources, sometimes overlapping other topics within the vast field of professional career interventions. The Self-Perceived Employability Scale has potential applications in workers’ career counselling and career coaching, and in undergraduates’ and graduates’ vocational guidance; applied to the workers within the organization or to the wider population, the scale could be included in the strategies to implement individuals in responding flexibly to changing circumstances inside and outside the organization [101]. Furthermore, for employees, the organizations could offer competency development initiatives for enhancing their employability perceptions, and through this, also for their feelings of career satisfaction and beliefs in their own marketability [152].

The results of our study, however, should be read in light of its limitations. First of all, the use of research cross-sectional design does not allow to verify the predictive validity of the scale; secondly, the convenience sampling poses limitations to the generalizability of the results to the population; the third methodological limitation is characterized by the use of only one sample to evaluate the scale structural validity: the verification of the instrument’s psychometric properties should be conducted using further independent samples. Therefore, future research could aim to replicate the present findings with a different sample. Finally, future works could demonstrate the factor invariance of the instrument in diverse samples, and in employing cross-cultural samples, future research will have to verify if the actual findings could be generalized to different cultural groups.

## Figures and Tables

**Table 1:** Items’ descriptive statistics.

Items	Mean	SD	Skewness	Kurtosis
Item 1	3.47	1.22	−0.60	−0.48
Item 2	3.31	1.19	−0.49	−0.58
Item 3	3.36	1.09	−0.56	−0.27
Item 4	3.83	1.06	−0.94	0.35
Item 5	3.67	1.09	−0.77	0.07
Item 6	3.49	1.04	−0.55	−0.07
Item 7	3.98	0.86	−0.98	1.57
Item 8	3.31	1.18	−0.42	−0.63
Item 9	3.31	1.16	−0.33	−0.66
Item 10	3.44	1.04	−0.44	−0.23
Item 11	3.16	1.05	−0.19	−0.31

**Table 2:** Exploratory Factorial Analysis with factorial loadings ^1^.

Items	External Work Opportunities	Ability Evaluation	Future Perspective
Item 9	0.901		
Item 8	0.887		
Item 10	0.599		
Item 11	0.436		
Item 5		0.951	
Item 4		0.530	
Item 6		0.509	
Item 3			0.770
Item 2			0.586
Item 1			0.530

^1^ Factor loadings < 0.25 are not showed.

**Table 3:** Factors’ descriptive statistics.

Factors	Mean	SD	Skewness	Kurtosis	Cronbach’s Alpha
Future Perspective	10.16	2.72	−0.53	−0.01	0.67
Ability Evaluation	10.99	2.56	−0.71	0.34	0.72
External Work Opportunities	13.23	3.60	−0.44	−0.12	0.83

**Table 4:** Correlations between Self-Perceived Employability Scale, Career Adaptability sub-dimensions and Well-Being indices.

Employability Dimensions	Career Concern	Career Curiosity	Career Confidence	Career Control	Life Satisfaction	Flouris-Hing	Job Satisfaction
Future Perspective	0.182 **	0.150 **	0.172 **	0.153 **	0.372 **	0.349 **	0.469 **
Ability Evaluation	0.241 **	0.236 **	0.227 **	0.201 **	0.203 **	0.296 **	0.205 **
External Work Opportunities	0.141 **	0.153 **	0.142 **	0.125 **	0.256 **	0.308 **	0.226 **

** *p* < 0.001.

**Table 5:** Regression of Self-Perceived Employability on Well-being indices.

Dependent Variable	Predictors	β	t	*p*	Model Statistics
Life Satisfaction	Future Perspective	0.32	7.96	<0.001	R^2^ = 0.15 F = 39.90 (*p* < 0.001)
	Ability Evaluation	NS	NS	NS	
	External Work Opportunities	0.13	2.97	0.003	
Flourishing	Future Perspective	0.25	6.21	<0.001	R^2^ = 0.17 F = 44.75 (*p* < 0.001)
	Ability Evaluation	0.13	3.00	0.003	
	External Work Opportunities	0.15	3.57	<0.001	
Job Satisfaction	Future Perspective	0.45	11.66	<0.001	R^2^ = 0.22 F = 62.83 (*p* < 0.001)
	Ability Evaluation	NS	NS	NS	
	External Work Opportunities	NS	NS	NS	

## Appendix A

**Table A1:** Italian adaptation/English original version of Self-Perceived Employability Scale.

Items
1. Se in questa organizzazione ci fossero dei licenziamenti, ho fiducia nella mia permanenza/Even if there was downsizing in this organization, I am confident that I would be retained.
2. La rete dei miei rapporti personali in questa organizzazione aiuta la mia carriera/My personal networks in this organization help me in my career.
3. Sono consapevole delle opportunità che in prospettiva potrei avere in questa organizzazione anche se fossero differenti da ciò che faccio ora/I am aware of the opportunities arising in this organization even if they are different to what I do now.
4. Le capacità che ho acquisito col mio attuale lavoro sono trasferibili ad altre occupazioni al di fuori di questa organizzazione/The skills I have gained in my present job are transferable to other occupations outside this organization.
5. Potrei facilmente riqualificarmi per rendermi impiegabile altrove/I could easily retrain to make myself more employable elsewhere.
6. Conosco le opportunità che avrei fuori da questa organizzazione anche se sono abbastanza differenti da ciò che faccio ora/I have a good knowledge of opportunities for me outside of this organization even if they are quite different to what I do now.
7. In questa organizzazione, le persone che fanno il mio stesso lavoro mi stimano/Among the people who do the same job as me, I am well respected in this organisation. ^1^
8. Se fosse necessario, potrei facilmente trovare un altro lavoro come il mio in una organizzazione simile/If I needed to, I could easily get another job like mine in a similar organization.
9. Potrei facilmente trovare un lavoro simile al mio in un’altra organizzazione/I could easily get a similar job to mine in almost any organization.
10. Chiunque con il mio livello di capacità, conoscenze ed esperienza, ha alte probabilità di essere richiesto da altri datori di lavoro/Anyone with my level of skills and knowledge, and similar job and organizational experience, will be highly sought after by employers.
11. Potrei ottenere qualsiasi lavoro e ovunque poiché le mie capacità e la mia esperienza sono rilevanti/I could get any job, anywhere, so long as my skills and experience were reasonably relevant.
12. La rete dei miei rapporti personali in questa organizzazione aiuta la mia carriera/My personal networks in this organization help me in my career.
13. Sono consapevole delle opportunità che in prospettiva potrei avere in questa organizzazione anche se fossero differenti da ciò che faccio ora/I am aware of the opportunities arising in this organization even if they are different to what I do now.
14. Le capacità che ho acquisito col mio attuale lavoro sono trasferibili ad altre occupazioni al di fuori di questa organizzazione/The skills I have gained in my present job are transferable to other occupations outside this organization.

^1^ item 7 was deleted in the Italian Adaptation.
